# Is Weekly Frequency of Yoga Practice Sufficient? Physiological Effects of Hatha Yoga Among Healthy Novice Women

**DOI:** 10.3389/fpubh.2021.702793

**Published:** 2021-10-18

**Authors:** Barbara Csala, Renáta Szemerszky, János Körmendi, Ferenc Köteles, Szilvia Boros

**Affiliations:** ^1^Doctoral School of Psychology, ELTE Eötvös Loránd University, Budapest, Hungary; ^2^Institute of Health Promotion and Sport Sciences, ELTE Eötvös Loránd University, Budapest, Hungary

**Keywords:** hatha yoga, BMI, flexibility, balance, core strength, heart rate, heart rate variability

## Abstract

Beneficial physical and physiological health outcomes of yoga practice are well-supported by empirical data. However, whether weekly frequency of training is sufficient to evoke positive changes, is still an open question. The present intervention study investigated the effects of 10 weekly sessions of beginner level hatha yoga with respect to indicators of physical fitness and physiological markers. 82 young women (mean age of 22.0 ± 3.83 years) participated in the study. The yoga group (*N* = 49) attended a yoga course consisting of 10 sessions (1.5 h each) on a weekly basis. The control group (*N* = 33) did not receive any intervention. BMI, body fat percentage, balance (one-leg-stand test with open and closed eyes, functional reach test), flexibility (side bend test, modified sit and reach test) core muscle strength (plank test) as well as resting heart rate (HR), and heart rate variability (HRV) were assessed 1 week before and after the course. Both frequentist and Bayesian analysis showed an improvement in flexibility and balance in the yoga group compared to the control group. The yoga group showed also increased core muscle strength. No changes with respect to BMI, body fat percentage, resting HR and HRV were found. Ninety minute beginner level hatha yoga classes were characterized by 93.39 HR and 195 kcal energy consumption on average. The present findings suggest that weekly setting of a 10-session long hatha yoga training leads to improvements in balance, flexibility and core muscle strength among healthy young women. However, for changes in BMI, body fat percentage, resting HR and HRV longer, and/or more intense interventions are needed.

## Introduction

Yoga is a complex philosophy and methodology which evolved over thousands of years in ancient India (1, 2). According to traditional definition as it is described in the famous Yoga Sutras of Patanjali, “Yogaschitta vrtti nirodhah” [(3), p. 31] which means that yoga is stopping the fluctuations/patterns of the mind. For a scientifically more clear definition of yoga, we can describe it as a path of self-realization which provides a holistic teaching, a complete view of life and a practical path for this sake (1, 4–9). Yoga comprises various physical, mental, moral, and spiritual practices which facilitate holistic health, well-being, and higher level of awareness (2, 4–10). There are various paths of yoga, however, the majority of the yoga classes offered in the West are body-focused practices which fall under the umbrella of hatha yoga (11). The practice of hatha yoga includes physical postures (asana), breathing exercises (pranayama), relaxation, and sometimes also meditation techniques (2, 12). It differs from other forms of physical exercise in many ways: it possesses specific body postures including relaxation poses, breath regulation, longer maintenance of the postures, furthermore, it requires constant non-judgmental attention during practice (4, 7, 13–15). Among these, breath regulation and constant awareness during practice are features of other mind-body exercises (16, 17) and other Eastern movement forms (such as aikido or karate) (18, 19).

During the last three decades, research on yoga has gained momentum, mainly due to its positive effects on health (17, 20, 21). Reviews and comparison studies indicate that regular yoga practice has beneficial effects on various aspects of mental and physical health (10, 13, 22–24). Yoga appears to be a valid method to improve physical and mental functioning in healthy populations, and it also offers therapeutic effects for a wide variety of pathological physical and psychological conditions (13, 22, 25, 26). Concerning physical fitness and cardiovascular characteristics, review studies mainly report positive outcomes in terms of flexibility, balance, strength, weight loss, blood pressure, respiration, heart rate, and overall cardiovascular endurance (10, 21–23, 27).

Regarding body weight and body mass index (BMI), a randomized controlled pilot study (28) investigated adults with high risk of diabetes. According to the outcomes, the yoga group showed reduced body weight and BMI at the end of the 8-week long (3–6 days/week, 75 min/session) intervention compared to the monitored walking group (28). Similarly, another study investigating healthy adults (29) reported a significant decrease in BMI after a 1-month long daily (1 h) yoga training compared to the control group which received no intervention. A recent study (30) found significant decrease in body weight, BMI, and body fat percentage after a 1 week daily (1.5 h) yoga practice among overweight adults. However, yoga practice was accompanied by dietary advisement. In contrast, Tran et al. (31) did not found a significant change in body fat among healthy young adults after an 8-week long (2 times/week, 1.5 h/session) yoga course.

A systematic review (32) summarized the effects of yoga practice on balance in healthy samples including participants from school children to elder adults. Out of 15 papers, 11 reported positive findings, especially in static balance. A later study (33) investigated middle-aged women participating in a power yoga program over 12 weeks (3 times/week, 1 h/session). Improvements in balance (both with open and closed eyes) and muscle (grip and back muscle) strength were found. Additionally, participants showed positive changes in body composition but no difference in BMI (33). Another study (34) investigated male college athletes. For 10 weeks, yoga participants attended two yoga classes per week (1 h/session) which were conducted before their usual sport training. The yoga group showed significant improvements in balance (measured by the stork stand test) and flexibility (assessed by the sit and reach test, shoulder flexibility, and joint angles measurements), while no significant changes in the control group were found (34).

Concerning further results on flexibility, middle-aged women showed a significant improvement in pliability (measured by the sit and reach test) after a 6-week long weekly (1.5 h/session) Iyengar yoga training (35). Betterment in flexibility (recorded by modified sit and reach test and trunk and neck extension test) were found also among adult male participants after a 12-week long (6 days/week, 1 h/session) hatha yoga training (36). In addition to the favorable changes in flexibility, increased muscle strength (grip and back), and decreased body weight and fat percentage were shown (36).

Beneficial effects of yoga practice on muscle strength were reported in some of the above-mentioned studies (22, 33, 36); however, core muscle strength was not investigated by any of them. A comprehensive review (37) examining the impact of yoga practice on core muscle strength indicated that yoga practice, more precisely some simple yoga exercises have a great relevance in strengthening core muscles. Kumar et al.'s results (38) showed a significant improvement in core muscle strength assessed by the plank test after a 21-day long hatha yoga training among healthy adults (38). Similar positive outcomes were reported in two recent studies (39, 40).

Regarding cardiovascular effects such as heart rate (HR) and heart rate variability (HRV) results are equivocal. HR and HRV (variable beat-to-beat fluctuation in HR) are sensitive and easily captured indicators of autonomic regulation and vagal activity. High frequency (HF) components of HRV are considered an index of parasympathetic activity, while low frequency (LF) components of HRV are often related to sympathetic activity. However, interpretation of LF band is controversial (41, 42). Bidwell et al. (43) found no significant differences in resting HR and HF and LF components of HRV between the yoga and the control group after a 10-week long (2 times/week, 1 h/session) hatha yoga intervention among women with asthma (43). In contrast, a systematic review (44) reported an evidence of decreased HR in both healthy and high risk populations based on studies with yoga interventions of diverse length and intensity. According to a comprehensive review on yoga and HRV (42), the vast majority of the studies investigated the acute effects of yoga practice on HRV, and indicated an increased HRV and vagal dominance (HF) during practice. Regular yoga practitioners showed increased vagal tone (HF) at rest compared to non-yoga practitioners. However, the authors advised not to draw a final conclusions on the impact of yoga on HRV (42). Two recent studies (45, 46), nevertheless, also reported positive outcomes. Breast cancer patients showed improved HRV values at end of a 12-month long (3 times/week, 40–60 min/session) yoga training. These results were also significantly better than those of a Pilates control group (46). Similarly, patients with rheumatoid arthritis showed improved HRV values after a 12-week long (3 times/week, 30 min/session) yoga practice compared to the only medical treatment control group (45).

As presented above, there is a growing body of empirical evidence regarding the beneficial physical health impacts of yoga practice. Nonetheless, due to the diversity in research, generalization and standardization of the reported findings is a challenging task (11, 17). Several authors have signified that heterogeneity of yoga interventions make the comparison of the outcomes and the exploration of the particular effects of specific components and aspects of yoga practice among various populations difficult (11, 17, 22, 47). Yoga interventions should be described in more detail with respect to dosage and frequency of practice, type and components of yoga, and duration and location (context) of practice. It is also suggested to include information about the yoga instructor, the presence or extent of home practice, and any other potential biases and disturbing factors (47). Studies that address these methodological issues would enhance the likelihood of developing more adequate prevention, intervention, and rehabilitation programs for various populations (10, 22, 48).

As presented above, most of the papers reported favorable outcomes concerning physical fitness and cardiovascular measures. Nevertheless, almost all of these studies involved yoga trainings with at least biweekly frequency, or often with (almost) daily practice. However, we have less accurate knowledge whether weekly frequency of yoga practice can result in improvements of physical fitness and heart rate characteristics among healthy adults. Previous studies involving one yoga class per week for 10 weeks showed significant improvements in mental health, physical and social functioning, and cognitive-behavioral performance among various adult samples (49–51). A recent study (52) with the same yoga intervention setting showed an increase in the right hippocampal density in healthy young adults when compared to active and passive controls. In an earlier study which applied a Pilates-training on a weekly basis for 10 weeks, Pilates participants showed improvements in flexibility, balance, and core- and abdominal muscle strength compared to no intervention controls (53). The authors proposed that a one-time per week training might be sufficient to evoke beneficial outcomes for healthy young women with a sedentary lifestyle.

According to a specific shortage of the empirical findings, aim of the present study was to investigate whether weekly frequency of beginner level hatha practice is sufficient to evoke beneficial changes in indicators of physical fitness and heart rate measures among healthy female adults. It was examined whether a 10-session long, one time/week yoga training (with an emphasis on asana practice) can positively effect on body mass index (BMI), body fat percentage, balance, flexibility, core muscle strength, resting heart rate, and heart rate variability. Additionally, we intended to explore the training effect characteristics, namely HR and used calories during practice, of an average beginner level hatha yoga class.

## Materials and Methods

### Participants

Beginner level hatha yoga training and courses for control participants were announced on the university's online registration platform. Only female students were eligible in order to avoid potential gender effects. Yoga groups included a maximum of 14 people. Control participants were aware of receiving no intervention; however, they were rewarded with credits for the participation and could become aware of their physical fitness during measurements, similarly to the yoga intervention participants. To avoid various confounding factors, the following inclusion criteria were applied for each group: no known psychiatric diagnosis; no prior experience with yoga; commitment to maintain current regular physical activity level and not to begin any new body focus related practices (such as relaxation or meditation) throughout the study. Also, in order to control the exact amount of yoga practice, approval of avoiding self-practice or any guided yoga techniques (e.g., recordings or videos) at home was needed. Overall, 115 students were enrolled in the study, 74 students in the yoga group and 41 participants in the control group. Due to drop-out, missing measurements or missing more than two yoga classes in case of the yoga group, the eventual sample included 82 participants (mean age: 22.0 ± 3.83 years). The yoga group consisted of 49 individuals (mean age: 21.49 ± 2.3 years), the control group comprised 33 participants (mean age: 22.75 ± 5.32 years). Average age and degree level (measured by the level of degree of the current studies) of the two groups were statistically comparable (age: *d* = −1.29; *p* = 0.204; degree: yoga: 1.20 ± 0.41, control: 1.36 ± 0.49; *d* = −1.55; *p* = 0.127). Average physical activity level in the previous 3 months (measured by a 5-point rating scale: 1 = no regular participation in physical activities, only occasionally; 3 = 2–3 h per week; 5 = competes in sports or has at least five high-intensity workouts per week) was also statistically comparable in the two groups (yoga: 1.84 ± 1.2, control: 2.24 ± 1.23 years; *d* = −1.48; *p* = 0.142). Students were mostly engaged in running, spinning, aerobic, and fitness training; none of the groups were physically inactive.

At the beginning of the program, informed consent form was signed by each participant. The research was permitted by the Research Ethics Committee of the university.

### Procedure

Data collection lasted for three semesters. After registration, participants received information via e-mail about the appointments for physical measurements and the conditions of attendance. Measurements were taken 1 week before and 1 week after the yoga course at the Institute of Health Promotion & Sport Sciences of the university. Plank test, measured at the beginning of the first and last yoga class, was an exception; therefore, these data were not available for the control group.

The yoga course encompassed 10 weekly sessions, 1.5 h each. Control participants did not receive any training or alternative intervention, they took part solely in the baseline and post-intervention measurements. Yoga classes had an emphasis on asana practice; all types of poses were included, namely sitting, standing, balancing poses, kneeling, forward and backward bends, supine and prone positions, and inversions. Classes always started with a short body and breath scan and ended with a relaxation practice of 8–10 min in Savasana (the lying corpse position). Scripts for the sessions were prepared according to two highly valued yoga books (2, 54). These were prepared before the course and strictly followed by the yoga instructor, namely a certified (RYT500) female yoga teacher (BC, one of the authors), throughout all groups and all semesters (see Appendix for class plan).

The complete study also included psychological measurements in which two yoga groups were differentiated. However, those did not differ in any aspects, thus were merged together. Results of psychological outcomes are reported in another paper (55).

### Measures

*BMI* was calculated from the assessed body weight and height data. Body weight and *body fat percentage* was measured with OMRON BF-511 body composition scale (OMRON Healthcare Group, Kyoto, Japan) using given height, gender, and age data.

Static balance was measured both with open and closed eyes by the *one-leg-stand (stork) test* (56).The task is to hold a stand on one foot with placing the heel of the opposite foot on the inside of the standing leg at knee level. The test lasts for 60 s while the number of errors, namely off-balance situations are recorded: the movement of the standing leg or loss of contact of the upper leg with the standing leg. The test was measured once with open and then with closed eyes. *Functional reach test* (FRT) (57) also assesses static balance. A maximal stable forward reach is needed to be performed from a fixed standing start position with one arm extending forward. Difference between the arm's length and the measured maximal forward reach (cm) is calculated. Better result of two trials is recorded.

Flexibility was investigated by the *side bend test* and the *modified sit and reach test*. The *side bend test* (58) measures pliability of the thoracic and lumbar part of the back. The starting pose is standing straight with shoulders, scapulae, and head touching the wall. The participant is asked to bend to one side keeping his/her hands straight at the thighs. Difference between the starting and ending point at the tip of the middle finger sliding down along the thigh is calculated in cm. There is one bend to each side, average of the two sides were calculated as a final variable. The *modified sit and reach test* (59) assesses the flexibility of hip region, involving the lower back and hamstring muscles. This modified version of the test aims to control for the variable ratio of the length of the limbs and the trunk. The test person sits with head, back, and shoulders at the wall, measurement box is placed to his/her feet. Legs are straight with hip apart. To measure the start position, the performer reaches out with hands placed on each other while the back and the head stay in touch with the wall. Then, the person bends forward at maximum level with sliding the hands on the top of the box. Difference between the starting point and the end reach at the tip of the middle finger is recorded (cm).

*Plank test* (60) measures the muscular endurance of core muscles. Participant's task is to hold a forearm plank pose keeping a straight body position. Only forearms and toes are supported on the ground. Aim is to hold the position as long as possible. Time was recorded in seconds with a maximal length of 120 s.

*Heart rate* and *heart rate variability* data were collected with Firstbeat Teambelt system from FirstBeat SPORTS Team Pack (Firstbeat Technologies Ltd., Jyväskylä, Finland) (61). A chest belt was attached to the participant ribcage who was asked to sit quietly without any movements. The belt includes two built-in electrodes and a wireless unit transmitting data to a receiver on a computer in real time. The sampling rate is 1,024 Hz. Resting heart rate data were collected for 5 min and analyzed by the Firstbeat Sports software. Two measures were extracted: resting heart rate, that is *rHR (bpm) and RMSSD (ms)*, i.e., the root mean square of successive differences of interval. It is considered an index of parasympathetic activation of heart (42, 62, 63).

Firstbeat Teambelt system was also used during yoga practice to measure participants average heart rate (*AvgHR*) and energy consumption (*kcal*) during the 90-min-long classes. An average from all participated classes was calculated as a final value for both variables.

### Data Analysis

Statistical analysis was conducted with the help of JASP v0.12.2 software (64). To test the homogeneity of the two groups at beginning, Welch d-tests were applied for all measures. The potential effect of the intervention was calculated by 2 × 2 mixed ANOVA (time × intervention) for each variable with the exception of plank test. For that, due to missing control data, paired sample *t*-test was applied. Respective effect sizes were calculated for all tests. The sample size varies across the variables due to lacking or invalid data at particular measures.

Beyond the conventional frequentist analysis, Bayesian tests were also conducted to shed more light on non-significant results. Bayes factor (BF_10_) shows the likelihood of an alternative hypothesis compared to the null hypothesis. If BF_10_ is smaller than 0.33, the null hypothesis is more probable than the alternative one. In contrast, BF_10_ above 10 indicates a strong support for the alternative hypotheses. BF_10_ values between 1 and 3 suggest only a weak or anecdotal indication for the alternative hypothesis (65). To avoid the well-known limitations of the classic frequentist statistics (those related to Type 1 and 2 errors), many authors have recommended the usage of Bayesian analysis as it provides a more precise answer to research questions compared to the frequentist one (65–67).

## Results and Discussion

### Results

Descriptive data of the measured physical and physiological variables in the two groups, and baseline comparisons are presented in Table 1. No significant differences between the groups emerged for any variables at baseline.

**Table 1:** Descriptive data of the assessed variables before and after the intervention, and baseline comparison of the groups.

			**Baseline**		**Baseline comparison (Welch-d)**			**Post-intervention**	
		***N***	**M**	**SD**	***D***	***df***	***p***	**M**	**SD**
BMI (kg/m^2^)	Y	49	21.59	2.72	1.01	63.61	0.318	21.53	2.71
	C	33	20.93	3.03				20.96	3.63
BodyFat%	Y	30	30.13	6.47	0.87	60.18	0.390	29.84	7.11
	C	33	28.73	6.35				28.33	6.35
One-leg-stand–Open eyes (NoE)	Y	49	0.14	0.50	0.99	73.84	0.325	0	0
	C	33	0.06	0.24				0.24	0.44
One-leg-stand–Closed eyes (NoE)	Y	49	4.65	3.78	0.48	74.32	0.632	3.82	3.27
	C	33	4.27	3.33				4.15	3.04
FRT (cm)	Y	47	41.07	6.03	−0.59	74.00	0.555	40.31	5.26
	C	33	41.65	5.48				40.22	5.67
Side bend (cm)	Y	49	20.64	5.11	−0.59	76.73	0.555	21.71	4.34
	C	33	21.25	4.21				19.02	3.79
Sit and reach (cm)	Y	46	33.01	6.91	−1.63	66.48	0.109	35.30	6.28
	C	33	35.33	7.44				33.43	6.91
Plank (sec)	Y	33	79.03	26.46	–	99.36	20.40		
rHR (bpm)	Y	42	83.22	11.74	0.44	58.07	0.660	84.77	11.58
	C	29	81.92	12.43				81.26	11.88
RMSSD (ms)	Y	42	40.68	22.51	−0.001	59.73	0.991	34.63	20.85
	C	29	40.75	22.87				36.17	18.67

*Y, Yoga; C, Control; NoE, Number of Errors; FRT, Functional reach test; rHR, resting HR*.

Results of the 2 × 2 mixed ANOVAs for the physical variables are presented in Table 2.

**Table 2:** Results of the 2 × 2 ANOVAs (both frequentist and Bayesian) for the physical variables.

		***F(df)***	***p***	*** $\eta_{p}^{2}$ ***	**BF_10_**
BMI	Group	0.890 (1,80)	0.348	0.011	0.651
	Time	0.009 (1,80)	0.924	< 0.001	0.170
	Group^*^time	0.076 (1,80)	0.783	< 0.001	0.288
BodyFat%	Group	0.790 (1,61)	0.378	0.013	0.794
	Time	1.792 (1,61)	0.186	0.029	0.433
	Group^*^time	0.041 (1,61)	0.839	< 0.001	0.298
One-leg-stand–Open eyes	Group	1.930 (1,80)	0.169	0.024	0.407
	Time	0.127 (1,80)	0.723	0.002	0.168
	Group^*^time	3.35 (1,80)	0.004^**^	0.099	17.807
One-leg-stand–Closed eyes	Group	0.001 (1,80)	0.972	< 0.001	0.255
	Time	1.397 (1,80)	0.241	0.017	0.417
	Group^*^time	0.779 (1,80)	0.380	0.010	0.318
FRT	Group	0.053 (1,78)	0.818	< 0.001	0.249
	Time	2.428 (1,78)	0.123	0.030	0.469
	Group^*^time	0.229 (1,78)	0.633	0.003	0.251
Side bend	Group	1.407 (1,80)	0.239	0.017	0.473
	Time	1.351 (1,80)	0.249	0.017	0.191
	Group^*^time	11.121 (1,80)	0.001^**^	0.122	22.828
Sit and reach	Group	0.021 (1,77)	0.884	< 0.001	0.415
	Time	0.179 (1,77)	0.673	0.002	0.285
	Group^*^time	20.779 (1,77)	< 0.001^***^	0.213	1029.277

** *p < 0.01.*

*** *p < 0.001. FRT, Functional reach test*.

There were no significant interactions for BMI, BodyFat%, one-leg-stand with closed eyes and FRT. In fact, Bayesian analysis showed the dominance of the null model for these variables. However, a significant interaction term emerged for one-leg-stand with open eyes, side bend test, and sit and reach test. Values of these three variables improved in the yoga group, while they declined in the control group. These outcomes were strongly supported by those of the Bayesian analysis with BF_10_ values over 10. In case of sit and reach, BF_10_ over 1,000 shows a saliently strong support. Concerning the plank test, paired sample *t*-test showed a significant result [*t*_(32)_ = −5.963; *p* < 0.001; Cohen's *d* = −1.038) which was also well-supported by the Bayesian analysis (BF_10_ = 14647.622).

Results of the 2 × 2 mixed ANOVAs for the physiological variables are presented in Table 3.

**Table 3:** Results of the 2 × 2 ANOVAs (both frequentist and Bayesian) for the cardiovascular variables.

		***F(df)***	***p***	*** $\eta_{p}^{2}$ ***	**BF_10_**
rHR	Group	0.888 (1,69)	0.349	0.013	0.455
	Time	0.118 (1,69)	0.732	0.002	0.200
	Group^*^time	0.738 (1,69)	0.393	0.011	0.317
RMSSD(ms)	Group	0.033 (1,69)	0.856	<0.001	0.275
	Time	3.859 (1,69)	0.054	0.053	1.177
	Group^*^time	0.074 (1,69)	0.787	0.001	0.264

*rHR, resting HR*.

No interaction effect emerged in resting HR and RMSSD; BF_10_ values indicated the superiority of the null hypothesis.

Finally, average HR and energy consumption during 90-min-long yoga classes showed the following results: *AvgHR* = 93.39 ± 9.78 bpm, min-max: 69.40–111.57; *kcal* = 195.83 ± 61.72, min-max: 76.63–348.71 (an average of all classes).

### Discussion

The present intervention study indicates that even a 10-session long beginner level hatha yoga training conducted on a weekly basis can result in improvements in balance, flexibility, and core muscle strength. However, it does not impact BMI, body fat percentage, resting heart rate and heart rate variability.

Studies which investigated BMI previously and reported a decrease in the outcomes involved a more frequent yoga training, e.g., daily yoga for 1 month (29), or 3-6 times/week training for 8 weeks (28). Average BMI of the participants in these two studies belonged to the overweight category (26.4 ± 2.5 and 28.4 ± 5.3, respectively). In contrast, the present intervention included a weekly setting of 10 yoga sessions, furthermore, participants had normal BMI at the beginning of the course. Thus, it can be suggested that changes in BMI may occur with more frequent than weekly practice, and/or after more than 10 sessions and supposedly among obese people. The latter assumption is also strengthened by the results of a systematic review (68). Besides, average energy consumption of a yoga class of the present investigation was 195 kcal (see later). Thus, we can also assume that for measurable changes in BMI greater exercise intensity and energy consumption are needed. Concerning body fat percentage, the present results did not show significant changes in this measure, either. This is line with the outcomes of Tran and colleagues (31) who similarly investigated young healthy adults and reported null findings. The above-mentioned review (68) also denotes the lack of effect of hatha yoga practice on body fat percentage compared to usual care, exercise, or lifestyle modification. In contrast, two studies reported a significant decrease in body fat percentage (30, 36). However, one of those (30) investigated overweight participants, and the other one (36) found significant changes in two elder subgroups (30–39 and 40–49 years) with higher than normal body fat percentage, but not in young participants (20–29 years) with normal body fat percentage. Furthermore, another study investigating the effects of a 12-week long (3 times/week) power yoga training reported significant decrease in body fat percentage among healthy middle-aged women (33). In summary, a decrease in body fat percentage might occur among elder and/or overweight participants with more intense yoga practice, but changes in body fat is not probable among healthy young adults after a short and less intense intervention.

Yoga participants of the present study showed a significant improvement in balance measured by the one-leg-stand with open eyes test compared to the control group. No changes measured by the one-leg-stand with closed eyes and FRT were found. Former studies (33, 34, 38) applying one-leg-stand with open or/and closed eyes reported significant betterment in balance, however they included more frequent and/or longer yoga programs. Similarly, the majority of the studies summarized by a systematic review (32) signified favorable outcomes, thus it can be concluded that 10 weekly sessions of yoga practice are sufficient to result in improvement of static balance with open eyes. However, for betterment in balance with closed eyes presumably longer or more intense yoga program might be needed. FRT, which similarly assesses static balance, can be rather noted as a measure of balance frailty (physical frailty) which shows moderate to strong negative association with age (69). Since the present investigation examined healthy young women, the differentiation of FRT might not be adequate enough among them to evaluate balance as a form of physical fitness. Nevertheless, it can also be proposed that 10 weekly session are not sufficient to register significant improvement in functional reach. Anyhow, improvement in balance through yoga in young ages is not only a sign of physical fitness but it can be a form of prevention of risks concerning falling in elderly ages (70).

Concerning flexibility, significant interaction effect in the sit and reach test and the side bend test showed that flexibility of yoga participants increased while it decreased in the control group at the end of the intervention. This is in line with the previous findings of Van Puymbroeck et al. (71). Compared to a light exercise group, improvements in the same flexibility tests were reported after an 8-week long (2 times/week) hatha yoga program (71). Other yoga studies investigating flexibility also found favorable outcomes in diverse male samples, like college athletes (34), preadolescent boys (72) and healthy men of various age groups (36). However, all of these studies (34, 36, 72) used more frequent interventions than the present one. According to our results, we can propose that even a weekly setting of yoga practice is sufficient to evoke improvements in flexibility. This is supported by Amin and Goddman (35) who found significant improvements in pliability with weekly sessions of yoga after 6 weeks. However, in that study (35) asanas were purposely selected to improve strength and flexibility.

Yoga participants showed increased core muscle strength measured by the plank test. Data of control participants were not available in this measure, however the large effect size and the high probability indicated by the Bayes Factor strongly support the significance of this change. Similarly, previous studies showed positive outcomes in core muscle strength after yoga interventions of various intensity (38–40). Two review articles (37, 73), furthermore, denoted the relevance of specific yoga postures for strengthening core muscles. It can be concluded that weekly regularity of yoga practice results in enhanced core muscle strength. Furthermore, diverse yoga poses together with yoga breathing induce various activation magnitudes of core muscles (included diaphragm that regulates intra-abdominal pressure and trunk stabilization), which is proposed to be considered by planning proper yoga interventions (73–75).

Regarding cardiovascular outcomes, the present study did not find significant changes in HR and HRV after the 10-session long intervention. As cardiovascular activation caused by the yoga classes was of slight intensity (see below), this finding is not surprising. Also, it is line with the findings of Bidwell et al. (43) who did not report differences in HR and HRV measures between the yoga and the control group after a 10-week long yoga training. In contrast, a systematic review (44) revealed evidence for effects of yoga on HR. However, with one exception of all the 19 included randomized controlled trials investigated longer and/or more frequent yoga programs. Concerning HRV, two recent studies (45, 46) also reported positive outcomes, however both applied longer yoga interventions. Thus, we can assume that although yoga may affect HR and HRV positively, 10 weekly sessions are not sufficient to evoke such changes.

According to the descriptive statistics of average heart rate and energy consumption, we can denote, that the average heart rate during beginner level hatha yoga classes (inclusive short body and breath focus, as well as an ~8–10 min lying relaxation) was 93.39 ± 9.78 bpm (min-max: 69.40–111.57), and average energy consumption was 195.83 ± 61.72 kcal (min-max: 76.63–348.71). Cowen and Adams (76) investigated heart rate during different styles of yoga classes of 80 min. They reported average heart rate during astanga yoga 95.18 ± 12.80, hatha yoga 80.17 ± 9.32, and gentle yoga 74.49 ± 7.41 bpm (76). Another study (77) reported 131 ± 14 bpm as average heart rate during a 45 min long power yoga class. A relatively recent study (78) showed that average heart rate of vinyasa yoga participant were 109.6 ± 18.0 bpm during a 60-min long class, and energy consumption were 273.8 ± 78.0 kcal. Compared to these previous results, yoga classes of the present intervention although beginner level, were around medium level in intensity among yoga styles.

It can be concluded that weekly setting of 10 beginner level traditional hatha yoga classes (1.5 h each) can result in significant positive changes in balance, flexibility, and core muscle strength in healthy female adults. However, it does not lead to changes in BMI, body fat percentage, and resting HR and HRV. Regarding BMI and body fat percentage, positive changes might be expected only in overweight population or with more intense yoga practices. Changes in resting HR and HRV supposedly occur only with longer and/or more intense yoga intervention. Concerning descriptive data of average heart rate and energy consumption during yoga classes, we can state that beginner level hatha yoga classes show medium intensity among yoga classes, and might be an appropriate form of exercise to meet some public health guidelines (78). All in all, we can conclude that a beginner level of hatha yoga program of 10 weekly sessions can result in beneficial physical health outcomes.

For future studies, we recommend the inclusion of various populations with the same yoga intervention setting to explore whether current results can be generalized across genders, different age and health groups. Besides, we propose to specifically investigate the advantageous effects of hatha yoga practices compared to other physical exercises. For that, the use of the terminology of gymnastics during yoga practice could be applied. This would provide a better understanding of yoga movements for those who are not familiar with yoga terminology (e.g., most coaches and sport scientists), furthermore, it could enhance the possibility to capture the exact effects of yoga exercises beyond terminology. However, for some specific aspects of yoga practice, such as particular poses, movements, gestures, breathing patterns, or attentional focus, this might be a difficult task. Nevertheless, the use of terminology of gymnastics in yoga could aid the proliferation of the beneficial physical effects of hatha yoga in such, that it might bring it closer to a wide circle of physical education teachers and sports professionals.

### Limitations

The sample of this study was restricted to young female participants. As further limitations we must mention the unbalanced sample size, the relatively large drop-out rate and lack of randomization of the design. However, to improve methodology, strict inclusion criteria for controlling potential confounding variables were applied (e.g., physical activity level in the previous months was measured; participants were not allowed to begin any new physical activities or body focus related activities beyond the current study; practicing yoga at home was not allowed). At the end of the intervention, participants were asked about their physical activity during the study period, however, it was not investigated by more precise measures. Future studies are proposed to control for this potential influencing factor with more accurate assessment, such as weekly activity tracking. Furthermore, we have to mention the lack of control data concerning plank test. During measurements, tests for interoception were also applied, among those the waterload test could have interfered with the usage of plank test. Thus, plank test was scheduled for the first and the last yoga classes. Lastly, it is relevant to mention that the control group did not received any intervention. However, main goal of the present study was not to assess the effectiveness of yoga practice compared to other physical activities, but to investigate whether weekly setting of a short beginner level yoga course can result in beneficial physical and physiological health outcomes. Nevertheless, attention received by the participants may have affected the results, thus present findings are restricted to face-to-face yoga practice, but might not be valid for home practice with recorded teachings. Potential biases during physical measurements before and after the training were minimized. Research assistants who conducted the measurements were blind to the group of the participants. However, motivations and expectations of participants were not monitored, which is suggested to do so in future studies.

## Conclusions

Present outcomes indicate that weekly setting of a beginner hatha yoga training of ten 1.5 h long sessions causes improvements in balance, flexibility, and core muscle strength, but does not affect BMI, body fat percentage, resting heart rate, and heart rate variability in healthy young women. It can be concluded that weekly frequency of yoga intervention is adequate for beneficial changes in certain aspects of physical fitness, however for betterment in physiological, especially cardiovascular makers higher weekly frequency and/or longer time of training is needed. The relevance of these findings accords with the conclusions of Tolnai et al. (53), that weekly training is beneficial for those who cannot afford more leisure time than this for physical activity. However, engagement in regular physical activity might increase over time as beneficial outcomes occur, and the process might be able to end in a virtuous circle.

## Data Availability Statement

The raw data supporting the conclusions of this article will be made available by the authors, without undue reservation.

## Ethics Statement

The studies involving human participants were reviewed and approved by Research Ethics Committee of the Faculty of Education and Psychology at ELTE Eötvös Loránd University, Budapest, Hungary. The patients/participants provided their written informed consent to participate in this study.

## Author Contributions

JK processed heart rate data. BC and FK processed all other data and performed the statistical analyses. BC wrote the first draft of the manuscript. RS suggested major improvements on it. All authors contributed to the assessment of data, read and commented on the last version of the manuscript, and conception and design of the study.

## Funding

This research was supported by the Hungarian National Scientific Research Fund (Orszs Tudomos Kutat Alapprogramok, OTKA K 124132).

## Conflict of Interest

The authors declare that the research was conducted in the absence of any commercial or financial relationships that could be construed as a potential conflict of interest.

## Publisher's Note

All claims expressed in this article are solely those of the authors and do not necessarily represent those of their affiliated organizations, or those of the publisher, the editors and the reviewers. Any product that may be evaluated in this article, or claim that may be made by its manufacturer, is not guaranteed or endorsed by the publisher.
