# Supplementary material for: Is Weekly Frequency of Yoga Practice Sufficient? Physiological Effects of Hatha Yoga Among Healthy Novice Women
Source: Front Public Health. 2021 Oct 18;9:702793. doi: 10.3389/fpubh.2021.702793 (PMC8559597; doi:10.3389/fpubh.2021.702793)
Supplement: Supplementary file 1 [file Table_1.DOCX]

**Appendix**

**Yoga class plan of the 10 yoga sessions in general (1.5 hours each)**

*Yoga classes always followed the same sequence regarding the groups of asanas, however sessions varied across which specific asana was/asanas were included from the particular group*

| **Group/Type of asanas** | **Sepcific asana(s)** |
| --- | --- |
| **Crossed leg seated position** | Sukhasana - inward focus, breath awareness *(always)* |
| **Warming up exercises** (Pawanamuktasana series) | movements of the joints: neck, wrist, elbow, shoulder, hip, knee, ankle - e.g.: Baddha Konasana *(5-7 short exercises)* |
| **Marjari asana** | cat-cow pose and its variations *(always)* |
| **Sunsalutation** | 12 steps:   1. Pranamasana 2. Urdva Hastasana 3. Uttanasana 4. Ashwa Sanchalanasana 5. Kumbhakasana 6. Asthanga Namaskara 7. Bhujangasana 8. Parvatasana 9. Ashwa Sanchalanasana 10. Uttanasana 11. Urdva Hastasana 12. Pranamasana   Hands and arms down, repetition with the other side.  *(1 round from class 3; 2 rounds from class 6)* |
| **Urdhva Mukha Svanasana, Adho Mukha Svanasana** | *(from class 2)* |
| **Standing poses** | Tadasana, Uttkatasana, Uttanasana, Virabhadrasana 1., Parsvottanasana, Virabhadrasana 2., Parsvakonasana, Trikonasana, Parivritta Trikonasana, Prasaritta Padottanasana *(3-6 of these poses)* |
| **Balancing postures** | Vriksasana, Garudasana, Hasta Padangustanasa beginner version, Ardha Chandrasana, Vhirabadrasana 3., Natarajasana *(1-2 of these poses)* |
| **Inversions *(only ocasionally)*** | Shasnakasna *(class 4)*, Ardha Sirsasana *(classes 7, 8)* Sirsasana *(classes 9, 10)* |
| **Prones poses** | Ardha Shalabasana, Shalabasana variations, Bhujangasana, Saral Bhujangasana, Kapotasana, Dhanurasana *(1-2 of these poses)* |
| **Forward bends and spine streches** | Balasana, Paschimottanasana, Janu Sirsasana, Upavista Konasana, Arda Matsyendrasana, Gomukasana *(1-2 of these poses)* |
| **Supine poses** | abdominal poses: Navasana and variations, Purvottanasana, Setu Bhandasana, Ustrsasana, Pawanamuktasana, Supta Hasta Pandagustasana, Chakrasana *(1-2 of these poses)* |
| **Inversions** | Sarvangasana, Halasana, Matsyasana *(1-3 of these poses)* |
| **Ending pose** | Spinal twist and/or Ananda Balasana *(always)* |
| **Savasana** | Relaxation for 8-10 minutes *(always)* |
